# Bis{4-[(3,5-dimethyl-1*H*-pyrazol-4-yl)selan­yl]-3,5-dimethyl-1*H*-pyrazol-2-ium} chloride monohydrate

**DOI:** 10.1107/S1600536812025640

**Published:** 2012-06-13

**Authors:** Maksym Seredyuk, Vadim A. Pavlenko, Kateryna O. Znovjyak, Elzbieta Gumienna-Kontecka, Larysa Penkova

**Affiliations:** a National Taras Shevchenko University, Department of Chemistry, Volodymyrska str. 64, 01033 Kyiv, Ukraine; b Faculty of Chemistry, University of Wroclaw, 14, F. Joliot-Curie Str., 50383, Wroclaw, Poland

## Abstract

In the title compound, 2C_10_H_15_N_4_Se^+^·Cl^−^·OH^−^, a singly protonated mol­ecule of the organic selenide participates in hydrogen bonding with neighboring mol­ecules, forming zigzag chains along [001]. The molecule adapts a *cis* bridging mode with a C—Se—C angle of 102.13 (15)°. π–π stacking inter­actions are observed between the closest pyrazole rings of neighboring chains [centroid–centroid distance = 3.888 (1) Å] and hydrogen bonding occurs through bridging chloride anions and hydroxide groups. Additionally, O—H⋯Cl hydrogen bonds are formed.

## Related literature

For details and applications of related pyrazoles, see: Krämer & Fritsky (2000 ▶); Fritsky *et al.* (2004 ▶); Kovbasyuk *et al.* (2004 ▶); Sachse *et al.* (2008 ▶); Penkova *et al.* (2009 ▶8). For structural studies of related bis­(1*H*-pyrazol-4-yl)selenides, see: Seredyuk *et al.* (2010*a* ▶). For structural studies of *d*-metal complexes of bis­(3,5-dimethyl-1*H*-pyrazol-4-yl)selenide, see: Seredyuk *et al.* (2007 ▶, 2009 ▶, 2010*b* ▶).
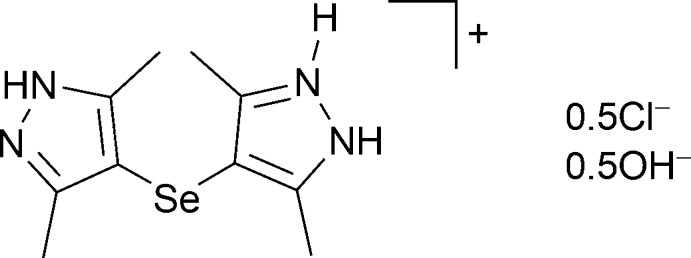


## Experimental

### 

#### Crystal data

2C_10_H_15_N_4_Se^+^·Cl^−^·HO^−^
*M* *_r_* = 592.90
Monoclinic, 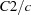

*a* = 22.805 (2) Å
*b* = 8.8154 (8) Å
*c* = 16.7462 (15) Å
β = 131.448 (7)°
*V* = 2523.4 (5) Å^3^
*Z* = 4
Mo *K*α radiation
μ = 3.07 mm^−1^
*T* = 100 K
0.25 × 0.20 × 0.12 mm

#### Data collection

Bruker SMART APEXII CCD diffractometer
Absorption correction: multi-scan (*SADABS*; Bruker, 2009 ▶) *T*_min_ = 0.488, *T*_max_ = 0.698
7656 measured reflections
2926 independent reflections
2211 reflections with *I* > 2σ(*I*)
*R*_int_ = 0.087

#### Refinement

*R*[*F* ^2^ > 2σ(*F* ^2^)] = 0.044
*wR*(*F* ^2^) = 0.107
*S* = 1.01
2926 reflections
160 parameters
H atoms treated by a mixture of independent and constrained refinement
Δρ_max_ = 1.15 e Å^−3^
Δρ_min_ = −0.72 e Å^−3^

### 

Data collection: *APEX2* (Bruker, 2009 ▶); cell refinement: *SAINT* (Bruker, 2009 ▶); data reduction: *SAINT*; program(s) used to solve structure: *SIR2004* (Burla *et al.*, 2005 ▶); program(s) used to refine structure: *SHELXL97* (Sheldrick, 2008 ▶); molecular graphics: *DIAMOND* (Brandenburg, 2009 ▶); software used to prepare material for publication: *SHELXL97*.

## Supplementary Material

Crystal structure: contains datablock(s) I, global. DOI: 10.1107/S1600536812025640/rk2354sup1.cif

Structure factors: contains datablock(s) I. DOI: 10.1107/S1600536812025640/rk2354Isup2.hkl

Supplementary material file. DOI: 10.1107/S1600536812025640/rk2354Isup3.cdx

Supplementary material file. DOI: 10.1107/S1600536812025640/rk2354Isup4.cml

Additional supplementary materials:  crystallographic information; 3D view; checkCIF report

## Figures and Tables

**Table 1:** Hydrogen-bond geometry (Å, °)

*D*—H⋯*A*	*D*—H	H⋯*A*	*D*⋯*A*	*D*—H⋯*A*
N1—H1*N*1⋯O1*W*	0.77	2.01	2.747 (3)	161
N4—H1*N*4⋯Cl1	0.77 (4)	2.42 (5)	3.146 (3)	160 (5)
O1—H1*O*⋯Cl1^i^	0.74	2.43	3.166 (4)	180
N2—H1*N*2⋯N3^ii^	1.03 (5)	1.78 (5)	2.804 (4)	177 (4)

Symmetry codes: (i) 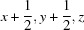; (ii) 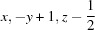.

**Fig. 1.**
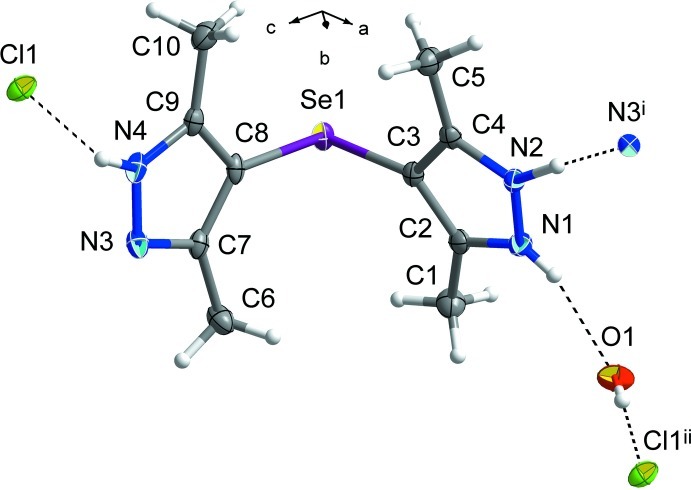
The title molecule with the atom numbering scheme. Displacement ellipsoids are drawn at the 50% probability level. Dashed lines show hydrogen bonds. Symmetry codes: (i) x, 1-y, -1/2+z; (ii) 1/2+x, 1/2+y, z.

**Fig. 2.**
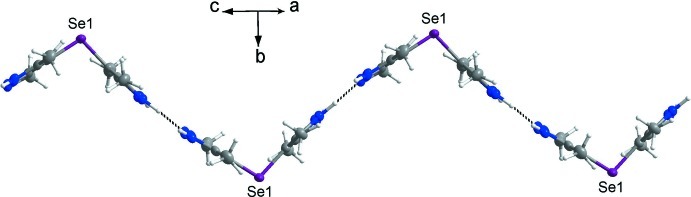
Zigzag chains of the organic selenide formed due to hydrogen bonding (dashed lines).

## supplementary crystallographic information

### Comment

Pyrazole-derived ligands are widely used in molecular magnetism, bioinorganic modelling and supramolecular chemistry due to their bridging nature and possibility for easy functionalization (Krämer *et al.*, 2000; Fritsky *et al.*, 2004; Kovbasyuk *et al.*, 2004; Sachse *et al.*, 2008; Penkova *et al.*, 2009). As a part of our synthetic and structural study of bis(1*H*-pyrazol-4-yl)selenides (Seredyuk *et al.*, 2010*a*) and their complexes with *d*-metals (Seredyuk *et al.*, 2007, 2009; Seredyuk *et al.*, 2010*b*), we report here the molecular and crystal structures of the title compound (Fig. 1).

In the cation of the title compound, a singly protonated molecule of the organic selenide (C_10_H_15_N_4_Se)^+^ participates in hydrogen bonding (*d*(N···N) = 2.804 (4)Å) with neighbor molecules forming zigzag chains along [0 0 1] (Fig. 2). The molecule adapts a *cis* mode of bridging with the C–Se–C angle of 102.13 (15)°. Between the closest pyrazole rings of the neighbor chains, π···π-stacking interaction is observed (centroid-centroid distance is 3.888 (1)Å) and hydrogen bonding through a bridging chloride anion (*d*(N···Cl) = 3.146 (3)Å) and a hydroxyde group (*d*(Ow···N) = 2.747 (3)Å). Additionally, a hydrogen bond Ow–H···Cl 3.166 (4)Å is found.

In the title compounds, the pyrazole rings exhibits C–C, C–N, N–N bond lengths which are normal for the substituted pyrazole molecules and close to those reported for related compounds.

### Experimental

A solution of a batch of bis(3,5-dimethyl-1*H*-pyrazol-4-yl)selenide (Seredyuk *et al.*, 2007)) in aqueous HCl_conc_ was disposed in a fridge at 277 K for one week. The obtained well formed colourless crystals were filtered off and air dried. C_10_H_17_ClN_4_OSe requires: C, 37.11; H, 5.29; N, 17.31. Found: C, 37.65; H, 5.37; N, 17.03.

### Refinement

The chlorine ion and the oxygen and hydrogen atoms of the hydroxide anion were found to occupy special positions (2-fold axis) with occupancy factors of 0.5. The H atoms from NH and OH were located from the difference Fourier map. The H atoms lined to N2 and N4 nitrogen atoms were refined freely, while hydrogen atoms of OH group and that linked to N1 nitrogen atom were constrained to ride on their parent atom, with *U*_iso_ = 1.5*U*_eq_(parent atom). The methyl H atoms were positioned geometrically and refined as riding atoms, with C–H = 0.96Å and *U*_iso_ = 1.5*U*_eq_(C).

### Crystal data

**Table:**

2C_10_H_15_N_4_Se^+^·Cl^−^·HO^−^	*F*(000) = 1200
*M**_r_* = 592.90	*D*_x_ = 1.561 Mg m^−^^3^
Monoclinic, *C*2/*c*	Mo *K*α radiation, λ = 0.71073 Å
Hall symbol: -C 2yc	Cell parameters from 3236 reflections
*a* = 22.805 (2) Å	θ = 3.3–28.3°
*b* = 8.8154 (8) Å	µ = 3.07 mm^−^^1^
*c* = 16.7462 (15) Å	*T* = 100 K
β = 131.448 (7)°	Block, colourless
*V* = 2523.4 (5) Å^3^	0.25 × 0.20 × 0.12 mm
*Z* = 4	

### Data collection

**Table:**

Bruker SMART APEXII CCD diffractometer	2926 independent reflections
Radiation source: fine-focus sealed tube	2211 reflections with *I* > 2σ(*I*)
Flat graphite crystal monochromator	*R*_int_ = 0.087
Detector resolution: 16 pixels mm^-1^	θ_max_ = 28.5°, θ_min_ = 3.3°
φ– and ω–scans	*h* = −30→30
Absorption correction: multi-scan (*SADABS*; Bruker, 2009)	*k* = −11→9
*T*_min_ = 0.488, *T*_max_ = 0.698	*l* = −21→21
7656 measured reflections	

### Refinement

**Table:**

Refinement on *F*^2^	Primary atom site location: structure-invariant direct methods
Least-squares matrix: full	Secondary atom site location: difference Fourier map
*R*[*F*^2^ > 2σ(*F*^2^)] = 0.044	Hydrogen site location: inferred from neighbouring sites
*wR*(*F*^2^) = 0.107	H atoms treated by a mixture of independent and constrained refinement
*S* = 1.01	*w* = 1/[σ^2^(*F*_o_^2^) + (0.0538*P*)^2^] where *P* = (*F*_o_^2^ + 2*F*_c_^2^)/3
2926 reflections	(Δ/σ)_max_ = 0.001
160 parameters	Δρ_max_ = 1.15 e Å^−^^3^
0 restraints	Δρ_min_ = −0.72 e Å^−^^3^

### Special details

**Table:**

Geometry. All s.u.'s (except the s.u. in the dihedral angle between two l.s. planes) are estimated using the full covariance matrix. The cell s.u.'s are taken into account individually in the estimation of s.u.'s in distances, angles and torsion angles; correlations between s.u.'s in cell parameters are only used when they are defined by crystal symmetry. An approximate (isotropic) treatment of cell s.u.'s is used for estimating s.u.'s involving l.s. planes.
Refinement. Refinement of *F*^2^ against ALL reflections. The weighted *R*-factor *wR* and goodness of fit *S* are based on *F*^2^, conventional *R*-factors *R* are based on *F*, with *F* set to zero for negative *F*^2^. The threshold expression of *F*^2^ > σ(*F*^2^) is used only for calculating *R*-factors(gt) *etc*. and is not relevant to the choice of reflections for refinement. *R*-factors based on *F*^2^ are statistically about twice as large as those based on *F*, and *R*-factors based on ALL data will be even larger.

### Fractional atomic coordinates and isotropic or equivalent isotropic displacement parameters (Å^2^)

**Table:**

	*x*	*y*	*z*	*U*_iso_*/*U*_eq_	
Se1	0.77024 (2)	0.12139 (4)	0.23905 (3)	0.01783 (13)	
Cl1	0.5000	0.43772 (14)	0.2500	0.0219 (3)	
O1	1.0000	0.5786 (4)	0.2500	0.0268 (9)	
H1O	1.0000	0.6625	0.2500	0.040*	
N1	0.86540 (17)	0.4303 (3)	0.1726 (2)	0.0181 (7)	
H1N1	0.8971	0.4766	0.1798	0.027*	
N2	0.78857 (17)	0.4481 (3)	0.0854 (2)	0.0161 (6)	
N3	0.73588 (18)	0.3657 (3)	0.4133 (2)	0.0198 (7)	
N4	0.66123 (19)	0.3412 (4)	0.3203 (3)	0.0205 (7)	
C1	0.9500 (2)	0.2805 (4)	0.3390 (3)	0.0252 (9)	
H1A	0.9760	0.2174	0.3240	0.038*	
H1B	0.9430	0.2250	0.3815	0.038*	
H1C	0.9811	0.3691	0.3772	0.038*	
C2	0.8718 (2)	0.3275 (4)	0.2366 (3)	0.0169 (7)	
C3	0.7966 (2)	0.2779 (4)	0.1888 (3)	0.0151 (7)	
C4	0.7459 (2)	0.3561 (4)	0.0927 (3)	0.0155 (7)	
C5	0.6585 (2)	0.3489 (4)	0.0064 (3)	0.0240 (9)	
H5A	0.6349	0.4060	0.0277	0.036*	
H5B	0.6417	0.2451	−0.0055	0.036*	
H5C	0.6429	0.3909	−0.0581	0.036*	
C6	0.8690 (2)	0.3080 (5)	0.4851 (3)	0.0251 (9)	
H6A	0.8830	0.3557	0.5472	0.038*	
H6B	0.8896	0.3660	0.4600	0.038*	
H6C	0.8902	0.2072	0.5027	0.038*	
C7	0.7813 (2)	0.3004 (4)	0.3993 (3)	0.0173 (7)	
C8	0.7356 (2)	0.2344 (4)	0.2981 (3)	0.0160 (7)	
C9	0.6587 (2)	0.2626 (4)	0.2498 (3)	0.0182 (8)	
C10	0.5831 (2)	0.2243 (4)	0.1410 (3)	0.0263 (9)	
H10A	0.5455	0.1925	0.1463	0.039*	
H10B	0.5916	0.1439	0.1111	0.039*	
H10C	0.5638	0.3122	0.0961	0.039*	
H1N4	0.629 (3)	0.372 (5)	0.319 (4)	0.032 (14)*	
H1N2	0.769 (2)	0.513 (5)	0.021 (4)	0.037 (12)*	

### Atomic displacement parameters (Å^2^)

**Table:**

	*U*^11^	*U*^22^	*U*^33^	*U*^12^	*U*^13^	*U*^23^
Se1	0.0250 (2)	0.01211 (19)	0.0227 (2)	0.00254 (15)	0.01847 (18)	0.00275 (15)
Cl1	0.0164 (6)	0.0200 (6)	0.0282 (7)	0.000	0.0143 (6)	0.000
O1	0.029 (2)	0.0157 (18)	0.024 (2)	0.000	0.0128 (19)	0.000
N1	0.0165 (15)	0.0195 (15)	0.0183 (16)	0.0001 (12)	0.0115 (14)	0.0006 (13)
N2	0.0172 (15)	0.0167 (15)	0.0171 (16)	−0.0006 (12)	0.0125 (14)	0.0005 (13)
N3	0.0199 (15)	0.0247 (17)	0.0175 (16)	0.0062 (13)	0.0135 (14)	0.0053 (13)
N4	0.0179 (16)	0.0260 (18)	0.0220 (18)	0.0056 (13)	0.0151 (16)	0.0079 (14)
C1	0.0207 (19)	0.031 (2)	0.022 (2)	0.0051 (16)	0.0131 (18)	0.0058 (17)
C2	0.0171 (18)	0.0192 (17)	0.0178 (19)	0.0003 (14)	0.0129 (16)	−0.0024 (15)
C3	0.0181 (18)	0.0131 (16)	0.0177 (19)	0.0009 (14)	0.0134 (16)	0.0007 (14)
C4	0.0212 (18)	0.0115 (17)	0.0172 (18)	0.0001 (14)	0.0142 (17)	−0.0010 (13)
C5	0.023 (2)	0.023 (2)	0.022 (2)	−0.0016 (16)	0.0137 (18)	0.0037 (16)
C6	0.023 (2)	0.033 (2)	0.018 (2)	0.0052 (17)	0.0126 (18)	0.0010 (17)
C7	0.0185 (18)	0.0176 (18)	0.0202 (19)	0.0046 (14)	0.0147 (16)	0.0070 (15)
C8	0.0215 (18)	0.0144 (17)	0.0195 (18)	0.0054 (14)	0.0167 (17)	0.0076 (14)
C9	0.0216 (18)	0.0177 (18)	0.023 (2)	0.0004 (14)	0.0178 (18)	0.0035 (15)
C10	0.021 (2)	0.031 (2)	0.028 (2)	−0.0020 (17)	0.0162 (19)	0.0033 (18)

### Geometric parameters (Å, º)

**Table:**

Se1—C8	1.904 (4)	C2—C3	1.398 (5)
Se1—C3	1.908 (3)	C3—C4	1.393 (5)
O1—H1O	0.7393	C4—C5	1.500 (5)
N1—C2	1.336 (5)	C5—H5A	0.9600
N1—N2	1.356 (4)	C5—H5B	0.9600
N1—H1N1	0.7666	C5—H5C	0.9600
N2—C4	1.333 (4)	C6—C7	1.504 (5)
N2—H1N2	1.03 (5)	C6—H6A	0.9600
N3—C7	1.333 (4)	C6—H6B	0.9600
N3—N4	1.363 (4)	C6—H6C	0.9600
N4—C9	1.338 (5)	C7—C8	1.400 (5)
N4—H1N4	0.77 (4)	C8—C9	1.384 (5)
C1—C2	1.498 (5)	C9—C10	1.499 (5)
C1—H1A	0.9600	C10—H10A	0.9600
C1—H1B	0.9600	C10—H10B	0.9600
C1—H1C	0.9600	C10—H10C	0.9600
			
C8—Se1—C3	102.13 (15)	C4—C5—H5B	109.5
C2—N1—N2	108.6 (3)	H5A—C5—H5B	109.5
C2—N1—H1N1	130.1	C4—C5—H5C	109.5
N2—N1—H1N1	121.3	H5A—C5—H5C	109.5
C4—N2—N1	109.4 (3)	H5B—C5—H5C	109.5
C4—N2—H1N2	127 (2)	C7—C6—H6A	109.5
N1—N2—H1N2	124 (2)	C7—C6—H6B	109.5
C7—N3—N4	105.0 (3)	H6A—C6—H6B	109.5
C9—N4—N3	112.4 (3)	C7—C6—H6C	109.5
C9—N4—H1N4	132 (4)	H6A—C6—H6C	109.5
N3—N4—H1N4	115 (4)	H6B—C6—H6C	109.5
C2—C1—H1A	109.5	N3—C7—C8	110.5 (3)
C2—C1—H1B	109.5	N3—C7—C6	120.6 (3)
H1A—C1—H1B	109.5	C8—C7—C6	128.9 (3)
C2—C1—H1C	109.5	C9—C8—C7	105.7 (3)
H1A—C1—H1C	109.5	C9—C8—Se1	126.3 (3)
H1B—C1—H1C	109.5	C7—C8—Se1	127.9 (3)
N1—C2—C3	108.2 (3)	N4—C9—C8	106.3 (3)
N1—C2—C1	121.4 (3)	N4—C9—C10	122.1 (3)
C3—C2—C1	130.4 (3)	C8—C9—C10	131.5 (3)
C4—C3—C2	105.8 (3)	C9—C10—H10A	109.5
C4—C3—Se1	127.2 (3)	C9—C10—H10B	109.5
C2—C3—Se1	126.8 (3)	H10A—C10—H10B	109.5
N2—C4—C3	108.0 (3)	C9—C10—H10C	109.5
N2—C4—C5	121.5 (3)	H10A—C10—H10C	109.5
C3—C4—C5	130.5 (3)	H10B—C10—H10C	109.5
C4—C5—H5A	109.5		
			
C2—N1—N2—C4	−0.7 (4)	Se1—C3—C4—C5	4.3 (6)
C7—N3—N4—C9	0.4 (4)	N4—N3—C7—C8	−0.2 (4)
N2—N1—C2—C3	0.0 (4)	N4—N3—C7—C6	178.2 (3)
N2—N1—C2—C1	178.8 (3)	N3—C7—C8—C9	−0.1 (4)
N1—C2—C3—C4	0.7 (4)	C6—C7—C8—C9	−178.3 (4)
C1—C2—C3—C4	−178.0 (4)	N3—C7—C8—Se1	−177.7 (2)
N1—C2—C3—Se1	175.3 (3)	C6—C7—C8—Se1	4.1 (6)
C1—C2—C3—Se1	−3.3 (6)	C3—Se1—C8—C9	99.9 (3)
C8—Se1—C3—C4	−82.7 (3)	C3—Se1—C8—C7	−83.0 (3)
C8—Se1—C3—C2	103.8 (3)	N3—N4—C9—C8	−0.5 (4)
N1—N2—C4—C3	1.1 (4)	N3—N4—C9—C10	−178.8 (3)
N1—N2—C4—C5	−178.9 (3)	C7—C8—C9—N4	0.3 (4)
C2—C3—C4—N2	−1.1 (4)	Se1—C8—C9—N4	178.0 (3)
Se1—C3—C4—N2	−175.7 (2)	C7—C8—C9—C10	178.5 (4)
C2—C3—C4—C5	178.9 (4)	Se1—C8—C9—C10	−3.9 (6)

### Hydrogen-bond geometry (Å, º)

**Table:**

*D*—H···*A*	*D*—H	H···*A*	*D*···*A*	*D*—H···*A*
N1—H1*N*1···O1	0.77	2.01	2.747 (3)	161
N4—H1*N*4···Cl1	0.77 (4)	2.42 (5)	3.146 (3)	160 (5)
O1—H1*O*···Cl1^i^	0.74	2.43	3.166 (4)	180
N2—H1*N*2···N3^ii^	1.03 (5)	1.78 (5)	2.804 (4)	177 (4)

Symmetry codes: (i) *x*+1/2, *y*+1/2, *z*; (ii) *x*, −*y*+1, *z*−1/2.
